# Mining Drug-Target Associations in Cancer: Analysis of Gene Expression and Drug Activity Correlations

**DOI:** 10.3390/biom10050667

**Published:** 2020-04-25

**Authors:** Monica M. Arroyo, Alberto Berral-González, Santiago Bueno-Fortes, Diego Alonso-López, Javier De Las Rivas

**Affiliations:** 1 Bioinformatics and Functional Genomics Group, Cancer Research Center (CiC-IMBCC, CSIC/USAL/IBSAL), Consejo Superior de Investigaciones Científicas (CSIC) and University of Salamanca (USAL), 37007 Salamanca, Spain; aberralgonzalez@usal.es (A.B.-G.); sjbuenofortes@usal.es (S.B.-F.); diego.alonso@usal.es (D.A.-L.); 2 Department of Chemistry, Pontifical Catholic University of Puerto Rico (PCUPR), 00717 Ponce, Puerto Rico

**Keywords:** cancer, cancer drug, drug activity, drug target, gene expression, transcriptomics, bioinformatics, correlation, gene network, bipartite network

## Abstract

Cancer is a complex disease affecting millions of people worldwide, with over a hundred clinically approved drugs available. In order to improve therapy, treatment, and response, it is essential to draw better maps of the targets of cancer drugs and possible side interactors. This study presents a large-scale screening method to find associations of cancer drugs with human genes. The analysis is focused on the current collection of Food and Drug Administration (FDA)-approved drugs (which includes about one hundred chemicals). The approach integrates global gene-expression transcriptomic profiles with drug-activity profiles of a set of 60 human cell lines obtained for a collection of chemical compounds (small bioactive molecules). Using a standardized expression for each gene versus standardized activity for each drug, Pearson and Spearman correlations were calculated for all possible pairwise gene-drug combinations. These correlations were used to build a global bipartite network that includes 1007 gene-drug significant associations. The data are integrated into an open web-tool called GEDA (Gene Expression and Drug Activity) which includes a relational view of cancer drugs and genes, disclosing the putative indirect interactions found for FDA-approved drugs as well as the known targets of these drugs. The results also provide insight into the complex action of pharmaceuticals, presenting an alternative view to address predicted pleiotropic effects of the drugs.

## 1. Introduction

Cancer is a leading cause of death worldwide, accounting for an estimated 9.6 million deaths in 2018 [1]. The most frequent cancers in the world in 2018 were lung and breast cancers, each adding 12.3 percent of the total number of cases diagnosed. Colorectal cancer was the fifth most prevalent cancer, with 1.8 million new cases in 2018 [2]. The five-year survival level for all cancer types has increased significantly from an average of 30% to 70% since the mid-1960s. Improvements in survival have been driven primarily by progress in early diagnoses and new drug therapies [1]. Survival is still profoundly affected by tumor metastasis and drug resistance [3,4]. Advance in cancer patient survival would be accelerated if these secondary malignant events (metastasis and resistance) could be overcome. In this regard, the identification of the molecular targets of each drug and the discovery of new drug targets in cancer are essential to improve the effectiveness of tumor therapy. The present work focuses on this objective, because, in many cases we still do not know what are the specific and the alternative targets of many drugs and pharmacologically active compounds.

Another relevant issue to cancer drugs and targets is the fact that the use of known and approved drugs for new medical indications has gained considerable momentum in the last decade [5]. Compared with the de novo drug discovery method, this strategy offers a significant benefit by decreasing price and time [6]. Drug repurposing, also referred to as drug repositioning, is a way to provide a new use for an approved therapeutic. Reused drugs have well-documented parameters of toxicity, pharmacology, and drug–drug interaction [7]. Therefore, drug repositioning in clinical studies costs only a small fraction of what is required to test a new drug in patients [8,9].

Pre-clinical biological models are necessary to record the molecular characteristics of cancer, including treatment reactions. Human cancer cell lines are a natural model. They are commonly used in drug development, such that the study of gene-drug interactions has been performed in multiple large-scale sensitivity screens [10]. The NCI-60 (60 human cancer cell lines used by the National Cancer Institute) includes information on pharmacological behavior for over 21,000 compounds, along with a broad spectrum of data on molecular profiling (such as gene expression and protein expression). The database Genomics of Drug Sensitivity in Cancer (GDSC) in the Sanger Institute [11] and the Cancer Cell Line Encyclopedia (CCLE) in the Broad Institute [12] are open data resources that focus on clinically appropriate drug activity information across many cell lines, with molecular profiling corresponding to the NCI-60 and clinical genomic studies. The Cancer Therapeutics Response Portal (CTRP) offers information on drug activity for almost 500 compounds in cell cultures that covers most of the data included in CCLE and GDSC. Each of these gateways permits an extensive search of its related datasets. Unfortunately, they do not allow an analysis of cross-databases, even though there is a significant overlap between cell lines and drugs [13].

Biomedical and clinical scientific knowledge about the specific molecular targets of many drugs has been recorded in several relevant databases. Probably the best known and most widely used resource reporting drug-target interactions is DrugBank [14]. More specific resources that include the molecular interactions of drugs and targets are PharmacoDB [15] and the Therapeutic Target Database (TTD) [16]. Several large studies have been publicly released testing candidate molecules, often with the corresponding molecular profiles of the cell lines used for drug screening. These studies are invaluable resources that allow researchers to leverage the data to perform new investigations [15]. One of the resources that best facilitates the retrieval and integration of the molecular and pharmacological data obtained for the NCI-60 cell lines is CellMiner [17,18,19]. These types of data can be used to search for protein activation or inhibition in different cell lines, as well as to explore the effect of drugs tested [20]. For example, this analytical approach was used to correlate the expression of EGFR and ERBB2 genes and the activity of the Erlotinib drug [21], as well as ABCB1 expression and Doxorubicin activity [18].

On the basis of the data resources described above, which include gene expression profiles and drug activity profiles, we postulated that, by making a selection of cancer drugs as well as a selection of cancer genes, it would be possible to find consistent significant correlations that link together drugs and targets. This is the primary goal of our work that undertakes a systematic approach to the discovery of connections among drugs and genes. These associations do not measure the direct molecular interaction of a drug with a protein target. Therefore, we should expect that, most of the time, these correlations reveal indirect interactions. In light of this limitation, we think that the correlation analysis described can provide a very interesting mapping of the gene products that can receive the effect, influence, or action of a given drug. These actionable targets proposed for a drug are what we defined as druggable modules. To achieve this mapping of drugs and associated genes, we needed the data produced for a collection of human cell lines where the activity of certain drugs was evaluated, together with the genome-wide expression of all human genes measured in the same collection of cells. The best collection of human cells, including this type of data, is the NCI-60 cancer cell lines. It is possible to calculate the correlation between the effect of one drug on the cell’s function and the expression of a given gene in the same cells using the data derived from these cancer cell lines. This correlation should be calculated robustly, estimating the *p*-value, and using several correlation coefficients. In this work, we performed these analyses and calculations for a collection of about one hundred Food and Drug Administration (FDA)-approved cancer drugs and a set of about four hundred cancer genes. The results provide an interesting compendium of significant pairwise correlations (drug–gene). These correlations were later used to build a bipartite network, where cancer drug–gene relationships can define interesting drug-target modules, and where specific clusters around critical nodes can be identified. Finally, with the results and information produced on drug-target interactions, we have developed an open web application tool called GEDA (Gene Expression and Drug Activity in cancer cells), where all our results can be freely accessed at: http://cicblade.dep.usal.es/GEDA/. This tool was developed using R and Shiny, and it allows the exploration of the significant correlations that we found between the cancer FDA-approved drugs and the human cancer genes. The data and results can be visualized using different types of correlation plots as well as selecting different interactive networks.

## 2. Materials and Methods

### 2.1. Data Resources Used: CellMiner, COSMIC, DrugBank, FDA-Approved Drugs

Data about the genome-wide expression of human protein-coding genes in the NCI-60 cancer cell lines were downloaded from the CellMiner database (v2.2) [16,18]. CellMiner is a pharmacogenomic resource that has the activity reports of over 20,503 chemical compounds, including 102 US FDA-cancer approved medications [17,18,19]. It also contains information about the activity or expression of 22,379 human genes, 92 proteins, and 360 microRNAs, as well as quantitative profiles of 10,350 proteins and 375 kinases derived from the NCI-60 data. The expression data from the human genes corresponded to mRNA normalized signal for each gene, converted to average *z*-score, taken from five combined gene expression platforms. These platforms were high-density oligo microarrays from Affymetrix: HG-U95, HG-U133, HG-U133 Plus 2.0, Human Exon 1.0 ST (Affymetrix Inc., Santa Clara, CA, USA), and from Agilent: Whole Human Genome Oligo array [22]. The processing and integration of these expression data and calculation of the *z*-scores have been previously described by Gmeiner et al. [22]. The complete expression data set (updated on 10 January 2017) contains expression values for 25,765 genes. For the drug activity data, we also downloaded from the CellMiner database the activity reports for 21,738 chemical compounds (updated 11 January 2017), including more than one hundred drugs approved by the U.S. Food and Drug Administration (FDA) for cancer treatment [18]. The activity corresponded to negative log10 (GI50) values of sulforhodamine B assay for each of the compounds tested in the NCI-60 cancer cell lines. The activity values were normalized and also converted to an average *z*-score [17,18].

The selection of cancer genes used in this study was taken from the Cancer Gene Census (CGC) data set (version 89, 15 May 2019), which is allocated in the Catalogue Of Somatic Mutations in Cancer (COSMIC) database [23,24]. A set of 705 cancer-related genes, common between CGC and CellMiner, was statistically analyzed as described below. Concerning the cancer drugs, the most recent FDA-approved cancer drug set (AOD IX, last updated 18 December 18) was obtained from the Developmental Therapeutics Program (DTP) from the Division of Cancer Treatment and Diagnosis (DCTD) [25]. This set contains 147 non-monoclonal antibody compounds approved for cancer therapy (i.e., FDA drugs). The complete collection of compounds tested was divided into two subsets: (i) FDA-approved cancer drugs, and (ii) all the other compounds not approved for cancer therapy (i.e., non-FDA drugs). The NSC (National Service Center) number was used as the unique identifier of each chemical compound.

The DrugBank database was used to find information on drugs and drug targets. DrugBank incorporates details of pharmaceutical and chemical data, including information about the specific known biomolecular targets of the drugs (reported as Pharmacologically Active drug against a given target). This information is beneficial to identify which of the predicted drug-target associations correspond to an experimentally proven pair, and also as a reference to propose the possible reuse of approved drugs for new treatment in different diseases [26]. DrugBank release version 5.1.4 (2 July 2019) was used to identify the known gene targets of the FDA-approved cancer drugs.

### 2.2. Statistical Analysis: Robust Calculation of Gene-Drug Correlations

The Hmisc R package [27] was used to calculate the *Pearson* and *Spearman* correlations between the gene expression z-scores and the drug activity *z*-scores, described above, across 60 cancer cell lines. First, the Pearson correlation between each gene-drug pair was calculated, and the results were filtered to select the correlations that had adjusted *p*-values ≤ 0.05 and correlation coefficients ≥ 0.334 in both cases (i.e., ≥0.334 for the Pearson’s *r* and the Spearman’s *rho* rank correlation coefficients). The adjustment for multiple testing of the *p*-values calculated for the correlations was done using Holm’s method, which asymptotically controls the error rate [27]; the correlation tests were also performed considering that the variables tested were not independent. The filters applied to the FDA-approved cancer drugs provided the set of positive associations (gene-drug links) selected. We then applied another filter for the drug activity, in order to avoid some compounds that gave a minimal signal in most of the cell lines tested. This filter eliminated the drug data when (i) the *z*-scores were not greater than 0.3 in at least 31 (31/60, 51.6%) of the cell lines (|0.3| in absolute values, positive or negative); or (ii) the *z*-scores were not >|1| (absolute values) in at least four cell lines. In the end, we obtained a set of 1007 correlations between cancer genes and FDA-approved cancer drugs. We also obtained a set of 6183 correlations between cancer genes and non-FDA drugs, although this second dataset is not presented in this work.

### 2.3. Construction of Gene-Drug Bipartite Networks

The correlation analysis described above generated sets of positive associations between drugs and genes that can be used to build bipartite networks. These types of networks are bipartite because they include associations between two types of nodes: the drugs (node type 1, the output of the interaction) and the genes (node type 2, the input of the interaction). The generation and visualization of the drug–gene networks were done using Cytoscape 3.7.2 [28]. In these networks, nodes represent genes and drugs, whereas edges represent the correlation between them. NetworkAnalyzer was used to analyze the topological properties of the network [29]. Several graph properties, such as degree and betweenness, were calculated and mapped to the attributes of the nodes or the edges. In the visualization, the nodes corresponding to genes were presented in different colors depending on their role in cancer: oncogenes, tumor suppressors, or others. Moreover, the correlation values were represented as the width of the edge.

### 2.4. Web Tool Presenting the Gene Expression and Drug Activity (GEDA): Correlations and Networks

R scripts were developed to generate gene expression and drug activity plots (barplots and scatterplots). In the barplots, the gene expression and the drug activity for each cell line are shown side by side. The cell lines are represented on the Y-axis, while the *z*-score value for the gene expression or the drug activity is represented on the X-axis. The bars for cell lines are marked (asterisk *) when they showed the same trend for expression and activity. A threshold of *z*-score > 1 and < −1 is marked with a vertical line to recognize significant changes more easily.

These R scripts were incorporated into a RStudio Shiny platform [30] to produce an open web tool called GEDA (Gene Expression and Drug Activity), which includes all the results presented in this work on cancer drug-gene correlations and networks. The web tool is accessible at: http://cicblade.dep.usal.es/GEDA/, in an interactive and downloadable format. In this website, all the significant correlations found between cancer genes and cancer drugs mentioned above, within the FDA and non-FDA datasets, can be selected. GEDA generates the barplots and scatterplots for the selected gene-drug pairs, as well as the data used to create the plots stored in a table (Data Table subsection) that can be downloaded by the user (including all of the values of the *z*-scores of each sample represented in the plots as well as other information about the samples). These plots and table are included in the section of the web tool called Correlations that also includes another subsection called Gene-drug Graphs, where each gene-drug pair is visualized in two graphs: (i) the Gene Graph, resenting all the interactions of a gene with drugs; and (ii) the Drug Graph, resenting all the interactions of a drug with genes. These graphs include a view of both the predicted associations of drug–gene (obtained in our study) plus the known and experimentally validated drug-target interactions (obtained from the DrugBank database). Another section on the GEDA website is called Networks, which includes an interactive visualization of the bipartite networks between cancer genes and drugs generated by GEDA. Finally, a section called Data includes two interactive tables: (i) one with detailed information about the currently known gene targets of the 92 FDA cancer drugs studied in our work (this information was also taken from the DrugBank database); and (ii) a second table including the 363 genes reported in this work. Several other R packages used for developing GEDA are described in the About section of the website.

## 3. Results and Discussion

### 3.1. Collection and Integration of Cancer Gene Expression and Drug Activity Data

The NCI-60 is a set of human cancer cell lines that includes 60 sample types corresponding to colorectal, kidney, ovarian, prostate, lung, breast, central nervous system, leukemia, and melanoma cell lines. CellMiner is a large, freely accessible data resource organized as a relational database that stores raw and normalized DNA, RNA, and protein, as well as pharmacological data produced using NCI-60 cell lines [17]. CellMiner has been previously used to study cancer drug activity (such as Afatinib, Erlotinib, Gefitinib, or Doxorubicin), as well as to elucidate cancer drug resistance. CellMiner has also been designed to allow broader pharmacogenomic studies [17,18,19]. The rcellminer R package was developed to allow researchers proficient in R to explore NCI-60 molecular profiling and drug activity data [31]. Unfortunately, in our experience, the datasets upon which rcellminer is dependent are not updated regularly, and the comparative analyses that this tool allows are limited. Considering this framework and the interest to achieve better associations between cancer drugs and their biomolecular targets, we downloaded all available data on gene expression and drug activity from NCI-60 cancer cell lines and developed our bioinformatic protocols to analyze such data.

**Figure 1:**
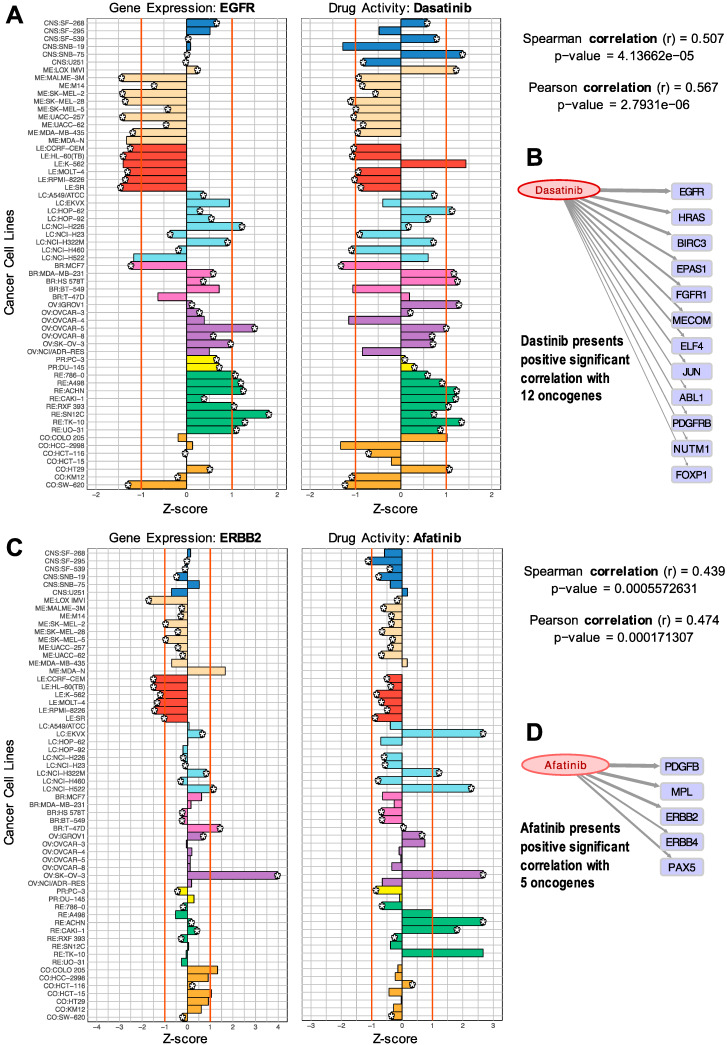
Comparison of gene expression versus drug activity (normalized profiles) to find significant correlations: (**A**) EGFR gene versus Dasatinib; (**B**) positive significant correlations of Dasatinib with 12 oncogenes; (**C**) ERBB2 versus Afatinib; (**D**) positive correlations of Afatinib with five oncogenes.

We started the analyses with 723 cancer genes from the CGC [23], of which 705 were found expressed in the cell lines, calculating the Pearson and Spearman correlations with 147 FDA-approved cancer drugs (as indicated in Materials and Methods). The results of this analysis provided a set of 1007 significant correlations that passed the thresholds and included 92 cancer drugs and 363 cancer genes. In order to visualize these results of the drug-target correlations, different graphics were used. Barplots (Figure 1A,C) were developed to present the profiles of the genes together with the profiles of the drugs. The gene expression and drug activity bars, corresponding to the *z*-score signal of the NCI-60 cancer cell lines, are plotted side by side to compare them. The plot shows gene-drug correlations with the same tendency in specific cell lines. An orange line marks *z*-scores at 1 and −1, to enhance the highest values (Figure 1A,C). Scatterplots presenting both *z*-scores in a two-dimensional space were also produced for each of the 1007 significant correlations.

The plots presented in Figure 1 facilitate the visual exploration of drug–gene target pairs found to be significant in the analysis of correlation. These pairs present predicted drug–gene associations measured by an indirect method (described above), providing an interesting mapping of the gene products that can receive the effect, influence, or action of a given drug.

The samples included in the barplots in Figure 1 present different colors to indicate different cancer subtypes and different tissue origins (i.e., breast, lung). The plots can be also useful to identify drug activity trends within a tissue type. Figure 1A shows the barplot of the expression of the EGFR gene and the activity of the drug Dasatinib. This drug is a well-known BCR-ABL1 tyrosine kinase inhibitor (TKI) used in the treatment of Philadelphia chromosome-positive adult acute lymphoblastic leukemia (Ph+ ALL) and chronic myelogenous leukemia (CML) [32,33]. The barplots in Figure 1A show that EGFR is expressed in lung, breast, ovarian, renal, and some colon cancer cell lines. Figure 1B shows all the oncogenes that were found with a significant positive correlation with Dasatinib. The thickness of the arrows is proportional to the Spearman correlation values. Some of these genes have been previously identified as drug targets, while the others are proposed as potential new candidate targets. 

It is important to recall that the method used to find these drug–gene associations does not measure the direct molecular interaction of a drug with a protein and, therefore, we should expect that most of the time, the correlations obtained will reveal indirect interactions. Knowing this limitation, the putative actionable targets associated with a drug can be used to define a druggable module. In many cases, we found references reporting studies that reveal the indirect action or effect of certain drugs on the non-canonical targets that we propose. For example, EGFR is not a known target of Dasatinib, but our analysis found this pair to have a significant correlation. Dasatinib has two known targets, ABL1 and PDGFRB [33,34], that were also found in our analyses. In fact, our results showed a Spearman correlation coefficient of 0.507 with EGFR, 0.355 with ABL1, and 0.356 with PDGFRB, respectively (all providing significant *p*-values). Thus, the correlation found of Dasatinib with EGFR could reveal a way of effective association. We found several studies that reveal the action of Dasatinib on EGFR. For example, the work by Sesumi et al., where the authors analyzed the effects of Dasatinib on the mechanism of resistance to EGFR TK inhibitors in lung cancer cells, showing that the combination treatment with Erlotinib and Dasatinib prevented the emergence of EMT (epithelial to mesenchymal transition) mediated resistance to EGFR inhibitors [35]. The positive effect of combining Erlotinib and Dasatinib for the treatment of non-small cell lung cancer (NSCLC), associated with the combined targeting of EGFR and SRC, has also been described in several clinical trials [36].

Moreover, a pharmaceutical mixture, including Dasatinib, has been efficient in inhibiting the expression of EGFR and BCL2L1 in the treatment of Glioblastoma tumors [37]. Finally, it has been shown that in EGFR-dependent NSCLC cell lines, the treatment with Dasatinib results in apoptosis. As a whole, Dasatinib is a potent multi-targeted inhibitor of SRC family kinases, BCR-ABL1, cKIT, PDGFRB, and Ephrin (EPH) receptors that have been shown to have anti-tumor effects in multiple solid tumors where EGFR overexpression is a common feature [38].

With respect to the effect of Dasatinib on FGFR1 (also reported in our analysis, Figure 1B), the sensitivity to Dasatinib as a tyrosine kinase inhibitor has been tested in vitro on an 8p11 myeloproliferative syndrome (using cell lines and primary cells of this rare tumor). Distinct sensitivity to Dasatinib was proven on these cells as compared with normal bone marrow control cells [39]. These cells presented translocations involving the fibroblast growth factor receptor 1 (FGFR1), as well as a BCR/FGFR1 fusion gene (confirmed and characterized by sequencing), indicating that Dasatinib somehow affects the cells where FGFR1 has specific alterations.

In the case of Afatinib (Figure 1C), the results showed a significant correlation with ERBB2 (a receptor tyrosine kinase, also known as HER2 or NEU), which is a critical oncogene involved in many adenocarcinomas (including breast and ovarian tumors, as well as lung). Afatinib is a potent and selective, irreversible ERBB family blocker. It is known that Afatinib covalently binds to and irreversibly blocks signaling from all homo- and heterodimers formed by the ERBB family members EGFR (ERBB1), HER2 (ERBB2), ERBB3, and ERBB4. In particular, DrugBank reports that Afatinib is pharmacologically active on ERBB2 and ERBB4. Figure 1D shows that Afatinib presented a significant positive correlation with five oncogenes: PDGFB, MPL, ERBB2, ERBB4, and PAX5. This result is consistent with the known action of this drug on ERBB2 and ERBB4, but it also provides three other candidates that are sensitive to Dasatinib.

The plots and drug–gene interactions presented in Figure 1 only correspond to two examples of the results produced in this work. To facilitate more in-depth exploration or particular queries on the results, all the data of the 1007 significant drug–gene correlations found in this study are included in Supplementary Table S1.

### 3.2. Identification of Known and New Potential Gene Targets for FDA-Approved Cancer Drugs

**Figure 2:**
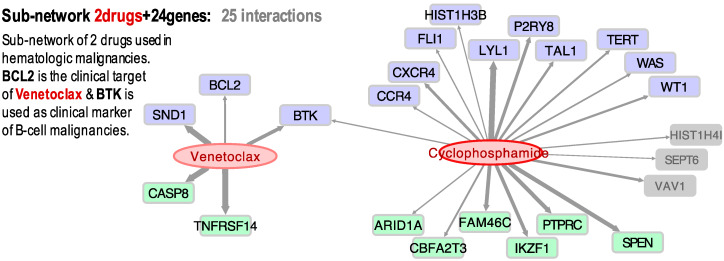
Bipartite directed sub-network of two cancer drugs (Venetoclax and Cyclophosphamine) and their target cancer genes: 13 oncogenes (blue); 8 tumor-suppressors (green); and 3 other cancer-related genes (grey) with an unassigned role. The links (grey arrows) show the significant correlation found between the expression profiles of the genes and the activity of the drugs, tested in cancer cell lines.

Figure 2 presents the significant correlations found for two drugs that are frequently used to treat hematologic malignancies: Venetoclax and Cyclophosphamide. We found that Venetoclax (NSC 766270) had a positive correlation with BCL2 (0.409). Venetoclax is a BCL2 selective inhibitor for the treatment of relapsed chronic lymphocytic leukemia (CLL) [40,41] and acute myeloid leukemia (AML) [42]. A recent study found that combination therapy of Ibrutinib and Venetoclax in patients with mantle-cell lymphoma was superior to monotherapy, with 71% of the patients showing a complete response [43]. Our results also showed significant activity (*z*-score > 4) in the leukemia HL-60(TB) cell line expressing caspase-8 (CASP8). CASP8 is considered a tumor suppressor, an initiator of apoptosis via the extrinsic pathway [44]. In this pathway, binding of the ligand to the receptor causes an intracellular switch, which promotes the activation of adapter proteins and the development of signals that promote cell death. Initiator caspases, such as caspase-8, are then cleaved, leading to the activation of downstream caspases that orchestrate apoptosis [45]. Interestingly, Venetoclax was previously shown to be caspase-dependent because the presence of the pan-caspase inhibitor Z-VAD-FMK inhibited the drug’s cell-killing activity [40]. Thus, our gene expression–drug activity correlation study is consistent with the previously shown relationship between BCL2-Venetoclax-CASP8 as a mechanistic molecular triad in the treatment of leukemia.

Our results also showed that Venetoclax and BTK have a Pearson coefficient of 0.468. Though BTK has not been identified as a molecular target for Venetoclax, two recent studies showed that therapeutic inhibition of the tyrosine kinase enhances the sensitivity of the drug in non-Hodgkin’s mantle cell lymphoma (MCL) and diffuse large B cell lymphoma (DLBCL) [46,47]. Cyclophosphamide (NSC 26271) was also found to have a positive correlation to BTK and LYL1 expression (Figure 2). This broad cancer spectrum drug has been extensively used for the treatment of various types of lymphoma and leukemia [48]. LYL1 is a transcription factor that is overexpressed in AML and may contribute to cell growth and drug resistance [49]. Given that our results show that Venetoclax and Cyclophosphamide share a common gene (BTK), we decided to explore if these two drugs have been used in combination therapy. Indeed, at present, there is a clinical phase I/II trial to study the effect of Venetoclax and Cyclophosphamide, as well as other chemotherapeutics, in patients with ALL (ClinicalTrials.gov; identifier: NCT03808610). 

Concerning the association of Venetoclax and BTK (Pearson = 0.46, Spearman = 0.45), although it is not a known drug-target canonical interaction, several recent experimental studies report some interaction. For example, Deng et al. suggested that BTK inhibition increased dependence and enhanced sensitivity of BCL2 to Venetoclax, supporting the trials combining BTK and BCL2 in chronic lymphocytic leukemia (CLL) [50]. Sasi et al. recently reported that pharmacological inhibition of BTK synergistically enhances Venetoclax sensitivity in BCL2+ cell lines [47]. Moreover, in a clinical trial, Jones et al. concluded that the activity of Venetoclax improved in CLL patients who were refractory to or who relapsed during or after therapy targeting BTK with Ibrutinib [51]. Finally, Tam et al. discovered that dual targeting of BTK and BCL2 with Ibrutinib and Venetoclax improved the outcomes in patients with mantle-cell lymphoma [43]. This shows that the networks and clusters generated from our gene expression and drug activity profiles may be used to identify new plausible interactions or drug-target associations that will help in the design and development of new therapies. 

**Figure 3:**
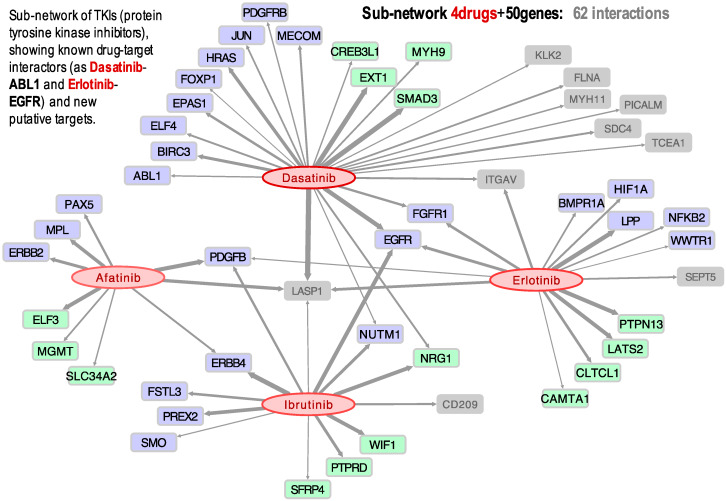
Bipartite directed sub-network of four drugs: Afatinib, Dasatinib, Erlotinib, and Ibrutinib, and their target cancer genes: 25 oncogenes (blue); 15 tumor-suppressors (green); and 10 other cancer-related genes (grey). The links (grey arrows) show the significant correlation found between the expression profiles of the genes and the activity of the drugs.

Afatinib, Dasatinib, Erlotinib, and Ibrutinib are drugs that belong to the family of tyrosine kinase inhibitors (TKIs), very much used in cancer treatment. In Figure 3, we present a small sub-network showing all the gene targets found in our study to have a significant correlation with these four TKIs.

The results indicate, for example, that the activity of three of these drugs (Dasatinib, Erlotinib, and Ibrutinib) presents a positive correlation with the expression of the gene EGFR, which is especially significant in lung and renal cancer (Figure 3). This observation is supported by a clinical trial published in 2014 in which a combination of Dasatinib and Erlotinib was successful in the treatment of non-small cell lung cancer (NSCLC) in patients with activating EGFR mutations [36]. Figure 3 further shows that Afatinib, Dasatinib, Erlotinib, and Ibrutinib have activities positively correlated to LASP1 expression, suggesting that this gene is a target of these four TKIs. Overexpression of LASP1 has been previously associated with tumor progression and metastasis in NSCLC, renal cell carcinoma (RCC), gastric cancer, and pancreatic cancer [52,53,54,55]. Another result found was the link between Dasatinib and PDGFRB, which had a Pearson correlation coefficient of 0.405. This drug-target link could be expected, because the mutation C843G in the PDGFRB protein has been identified to confer resistance to multiple ABL inhibitors, including Dasatinib. On the other hand, it has been reported that the presence of particular gene fusions, such as PDGFRB-EBF1, makes the patient an excellent candidate for treatment with TKIs [56].

Ibrutinib (NSC 761910) is an inhibitor of BTK prescribed for the treatment of lymphoma, leukemia, and B-cells cancers. Though Ibrutinib did not show activity in the leukemia cell lines included in CellMiner, it showed activity on the kidney cell lines, as well as some lung and ovarian cell lines. Furthermore, we detected a positive Pearson correlation between Ibrutinib and EGFR (Figure 3), most relevant in kidney, ovarian, and lung cancer cell lines. BTK is a non-receptor tyrosine kinase activated through the phosphorylation of a cysteine 481 (Cys481), triggering a signaling cascade that leads to cell differentiation, proliferation, and survival. EGFR is one of a few tyrosine kinases that have a homologous cysteine residue (Cys797), and Ibrutinib has been previously shown to inhibit mutant EGFR [57,58]. Moreover, it should be noted that Ibrutinib is being studied as a treatment in metastatic renal cell carcinoma (RCC), with successful results [59]. Our results also show that Ibrutinib presents a strong correlation with ERBB4 (Figure 3), which is another receptor tyrosine kinase of the same family as EGFR.

Afatinib is a selective inhibitor of the receptor tyrosine kinase ERBB2 (also known as HER2). The drug is approved for the treatment of NSCLC and has also been recently used to treat lung adenocarcinoma with mutated ERBB2. This tyrosine kinase is essential for molecular signaling as it is involved in cell proliferation, promotion of tumor growth, and resistance to apoptosis. Thus, it is an important oncogenic driver, with mutations identified in multiple types of cancer, including ovarian, bladder, and NSCLC [60]. Afatinib was found to have a rather significant positive correlation not only to ERBB2 expression (Figure 1C,D), but also to PDGFB (Figure 3). Platelet-derived growth factor-B (PDGFB) promotes proliferation and migration during angiogenesis, playing a role in tumor growth and metastasis in renal cancer cells, such that it has been proposed as a prognostic marker [61]. It can be seen in Figure 3 that the Afatinib, Erlotinib, and Ibrutinib activities are correlated to PDGFB expression. In fact, Ibrutinib was successfully used to treat a patient with RCC [59]. More recently, another patient was successfully treated for hereditary leiomyomatosis and renal cell carcinoma (HLRCC) with a combination of Bevacizumab and Erlotinib [62].

### 3.3. GEDA, Open and Accessible Web-Tool Including All Significant Drug-Target Correlations

In order to make our results accessible, a web-tool was developed, called GEDA (Gene Expression and Drug Activity). This web-tool allows one access to all the information about the positive drug-target correlations that we found in our study between cancer genes and FDA-approved cancer drugs, which includes a set of 1007 correlations. The tool presents the data into two types of plots: scatterplot and barplot. GEDA lets the user select from multiple options in order to create the plots, select the gene-drug correlation to the plot, and add or remove cell lines from the plot or highlight them.

**Figure 4:**
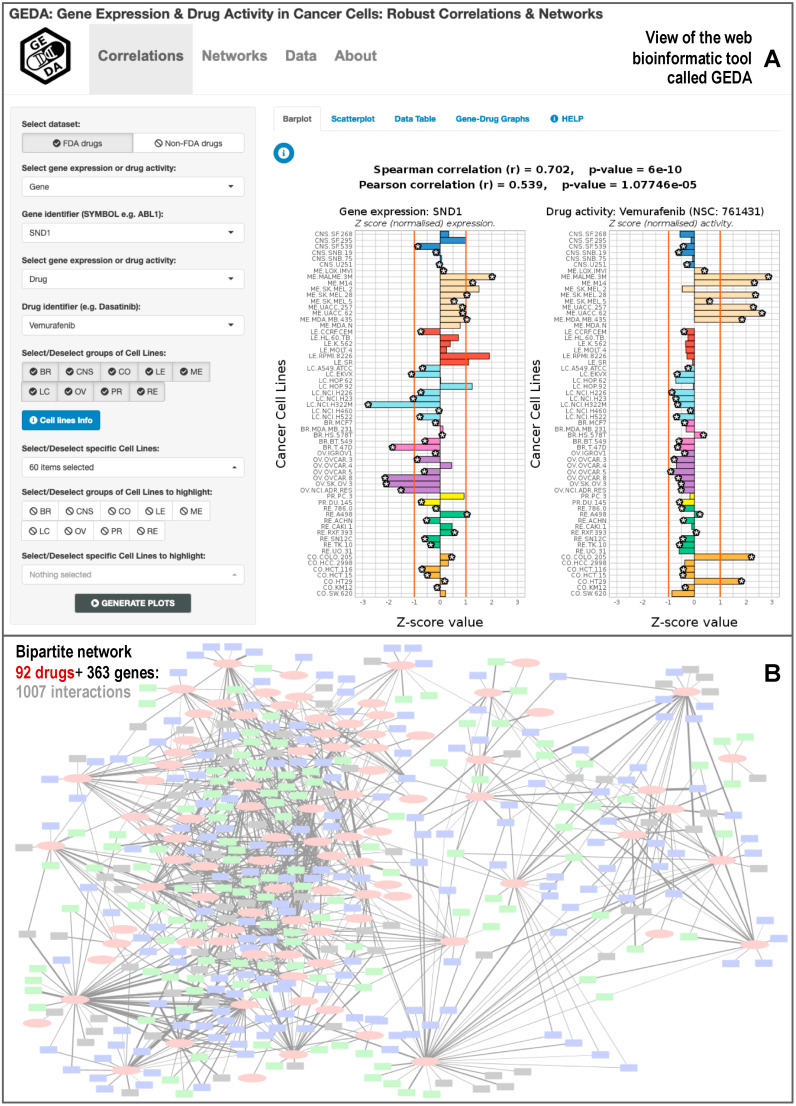
Bioinformatic web-tool called GEDA (Gene Expression and Drug Activity) that includes the database of significant correlations between cancer drugs and gene targets calculated using the information of 60 cancer cell lines (NCI-60). The resource presents (**A**) views of the data of each significant drug-target pair, using barplots and scatterplots; and (**B**) view of the bipartite networks generated between 92 Food and Drug Administration (FDA)-approved cancer drugs and 363 cancer human genes.

Figure 4 shows a view of the user interface and the barplots that can be generated with GEDA. The user can choose to plot the expression of a specific cancer gene and drug activity, selecting the names in the options panel on the left of the website. It is also possible to choose which cell lines to plot and highlight them for further study. The Pearson and Spearman correlation coefficients are provided, as well as the *p*-value corresponding to each coefficient. Additionally, GEDA includes bipartite networks presenting all the drug-target data produced in this work. Figure 4B shows a simplified schematic view of the entire network, which corresponds to 1007 correlations found between 363 cancer genes and 92 FDA-approved anticancer drugs. The networks can be viewed in GEDA and interactively explored as a whole, allowing the identification of possible small subnetworks or clusters of correlated drugs and genes. Interactive tables with all the genes and drugs present in GEDA included in the section called Data. Finally, any data chosen by the user to plot are put together in a table that can be exported. This table contains the gene expression, drug activity, tissue, and color used to plot it, as well as some metadata of the corresponding cell line. In future updates, we intend to add another dataset of drug-target correlations obtained for non-FDA drugs, which includes 6183 positive correlations. At present, this dataset is mentioned on the web.

Regarding the comparison of GEDA with other similar resources, we already mentioned in the introduction a series of tools that include transcriptomic and pharmacogenomic data derived from the analysis of cancer cell lines, such as CellMiner [17] and PharmacoDB [15]. CellMiner is the portal that best facilitates the retrieval and integration of the molecular and pharmacological data obtained for the NCI-60 cell lines, being a large pharmacogenomic resource that has activity reports of about 20,503 chemical compounds, including 102 US FDA-cancer approved medications [18,19]. CellMiner also contains information about the expression of 22,379 human genes, plus quantitative profiles of 10,350 proteins and 375 kinases derived from the NCI-60 data. PharmacoDB is another resource of particular relevance to this work, as it is a database focused on cancer drugs that also integrates multiple pharmacogenomic datasets based on cancer cell lines screening. In particular, PharmacoDB enables the analysis of public data sets to find previously profiled drug or cell lines, as well as dose-response data for a particular cell line [15]. Considering the size of these resources, GEDA is a much smaller tool focused on identifying significant correlations between anticancer drugs and cancer genes. Despite this limitation, as a whole, a description of the added value of GEDA to the field can be presented in three brief points: (i) GEDA is highly focused on a small set of cancer drugs and genes associations supported by correlation, with the aim of establishing a network view and allowing a better exploration of the complex relational space associated to the multiple interactions expected between genes and drugs; (ii) GEDA provides flexible graphical outputs of each gene-drug correlation found to be significant, and offers all the data available open to any user; and (iii) GEDA provides links and information, derived from Drugbank, to identify the known drug-target associations, but at the same time, is a data-driven resource that is not biased for a priori knowledge of specific drug-target interactions.

Finally, as indicated in several parts of this article, high throughput drug screening technologies have enabled the profiling of hundreds of cancer cell lines to a large variety of small molecules in order to discover novel and repurposed treatments [11]. Our findings in this work have shown that there is a great potential in exploring associations between pharmacological compounds and genes using large-scale omic data included in pharmacogenomic databases and resources.

## 4. Conclusions

Over the last two decades, there has been a shift in cancer treatment from cytotoxic and non-specific chemotherapeutics to targeted molecular treatments [63]. Despite this progress, in many cases, it remains necessary to identify and establish which are the biomolecular targets or mechanisms of action for many new pharmacological compounds introduced in the clinic. Further analyses may also assist in detecting unwanted off-target effects or identifying new functions of the target genes in a relational complex biological framework [64]. Our results show many plausible or expected cellular targets for FDA-approved cancer drugs, as well as new candidates derived from a thorough analysis of gene expression and drug activity. As such, our study may contribute to a better understanding of the mechanism of action of many drugs and present an alternative to exploring drug side-effects and drug resistance. Moreover, the development of the GEDA web-tool application may help researchers in identifying significant novel interactions and cancer gene-drug clusters to better understand the underlying molecular mechanisms of cancer therapy and drug action.

## Supplementary Materials

The following files are available online at https://www.mdpi.com/2218-273X/10/5/667/s1. Table S1: List of all of the 1007 significant correlations found between FDA-approved cancer drugs and cancer genes, including the details about the correlation parameters obtained for each drug-target pair.
